# Geldanamycin induces production of heat shock protein 70 and partially attenuates ototoxicity caused by gentamicin in the organ of Corti explants

**DOI:** 10.1186/1423-0127-16-79

**Published:** 2009-09-02

**Authors:** Yang Yu, Agnieszka J Szczepek, Heidemarie Haupt, Birgit Mazurek

**Affiliations:** 1 Molecular Biology Research Laboratory and Tinnitus Center, Department of Otorhinolaryngology, Charité - Universitätsmedizin Berlin, Campus Charité Mitte, Charitéplatz 1, 10117 Berlin, Germany; 2 Department of Otorhinolaryngology - Head & Neck Surgery, Tongji Hospital, Huazhong University of Technology & Science, Jiefang Ave. 1095#, 430030 Wuhan, PR China

## Abstract

**Background:**

Heat shock protein 70 (HSP70) protects inner ear cells from damage and death induced by e.g. heat or toxins. Benzoquinone ansamycin antibiotic geldanamycin (GA) was demonstrated to induce the expression of HSP70 in various animal cell types. The aim of our study was to investigate whether GA induces HSP70 in the organ of Corti (OC), which contains the auditory sensory cells, and whether GA can protect these cells from toxicity caused by a common aminoglycoside antibiotic gentamicin.

**Methods:**

To address these questions, we used the OC explants isolated from p3-p5 rats. As a read-out, we used RT-PCR, ELISA and immunofluorescence.

**Results:**

We found that GA at the concentration of 2 μM efficiently induced HSP70 expression on mRNA and protein level in the OC explants. Confocal microscopy revealed that HSP70 induced by GA is expressed by hair cells and interdental cells of spiral limbus. Preincubation of explants with 2 μM GA prior to adding gentamicin (500 μM) significantly reduced the loss of outer but not inner hair cells, suggesting different mechanisms of otoprotection needed for these two cell types.

**Conclusion:**

GA induced HSP70 in the auditory sensory cells and partially protected them from toxicity of gentamicin. Understanding the molecular mechanisms of GA otoprotection may provide insights for preventative therapy of the hearing loss caused by aminoglycoside antibiotics.

## Background

Heat shock proteins (HSPs) are a group of highly conserved proteins produced by all living organisms. HSPs maintain cellular homeostasis and are involved in the response to various stresses. The family of 70 kDa heat shock proteins (HSP70) are ubiquitously expressed HSPs. Under normal physiological circumstances, the constitutively expressed HSP70 (70 kDa heat shock cognate protein, HSC70) functions as chaperone during protein synthesis, intracellular transport and degradation. Under stress conditions, such as hyperthermia, oxidative stress, inflammation, exercise, exposure to toxins and ultraviolet light, starvation, hypoxia, or water deprivation, inducible HSP70 (iHSP70, HSP72) provide cellular protection by repairing the damaged proteins in cytoplasm and by blocking apoptosis [1,2]. Consequently, the heat shock proteins are also referred to as stress proteins and their up-regulation is sometimes described more generally as a part of stress response. The major stress-inducible isoform of HSP70 is encoded by two genes - *Hsp70-1 *(*Hspa1b*) and *Hsp70-2 *(*Hspa1a*) [3]. The expression of *Hsp70-1 *and *Hsp70-2 *is regulated *via *transcription factor heat shock factor 1 (HSF1). In cochlea, HSF1 is constitutively expressed in hair cells, spiral ganglion cells and in the stria vascularis [4,5]. Expressional induction of *Hsp70 *occurs in the inner ear (cochlea) in response to hyperthermia [6], transient ischemia [7], acoustic overstimulation [8] and cisplatin [9]. The otoprotective role of HSPs in the cochlea is well documented. Experiments with knock-out Hsf1^-^/^- ^and Hsp70^-^/^- ^mice, demonstrated that the expression of HSP70 is necessary to protect the cochlear hair cells from noise exposure and aminoglycoside ototoxicity [10,11].

Heat shock and pharmaceuticals are two known approaches of inducing HSP70 *in vivo*. Whole-body heat stress or a local heat shock (perfusion of hot saline into the middle ear cavity) substantially increased the expression of HSP70 in the cochlea and protected mice and guinea pigs from acoustic injury [12,13].

In clinical settings, local induction of HSP70 by pharmaceuticals is more feasible than by a heat shock. One of HSP70-inducing substances is geldanamycin (GA), a benzoquinone ansamycin antibiotic and antitumor drug, presently in clinical trials. GA binds to N-terminal ATP-binding site of HSP90, resulting in activation of HSF under non-stress condition [14]. GA has been shown to induce HSP70 expression and to provide protection in a number of mammalian cell types such as neurons, astrocytes and visual epithelial cells [15-18]. To date, however, there is no report on the auditory system, in terms of a protective effect of GA.

Gentamicin is an aminoglycoside antibiotic used to treat many types of bacterial infections, particularly those caused by Gram-negative bacteria. One of major side effects of gentamicin is ototoxicity [19]. The ototoxicity of gentamicin is attributed to the selective toxic effect on sensory hair cells in cochlea and vestibular organ. Although new generations of antibiotics have merged in the recent decades, aminoglycosides are still being used in a variety of medical conditions. Therefore, there are always considerable interests in finding ways to prevent their ototoxicity.

Our aim was to investigate whether GA induces the expression of HSP70 in the explant cultures of the organ of Corti (OC). Additionally, we wanted to test the ability of GA to protect the outer and inner hair cells (OHCs/IHCs) from ototoxicity caused by gentamicin.

## Methods

### Animals and dissection

Newborn Wistar rats (p3-p5) were used to prepare cochlear organotypic cultures. All studies were performed in accordance with the German Prevention of Cruelty to Animals Act and were approved by the Berlin Senate Office for Health (T0234/00).

The dissection procedure is similar to that described by Sobkowicz et al. [20]. After decapitation, the heads were cleaned with 70% ethanol and positioned with ventral surface down. The scalp was removed and the skull was transected along mid-sagittal plane. The brain was scooped out to expose the posterior fossa. The temporal bones were freed from the posterior hemi-skulls and transferred into Petri dishes containing cold, sterile, buffered saline glucose solution (BSG, glucose 11.4 mM). Under a stereomicroscope (Stemi, SV6, Zeiss, Germany), the tympanic membrane and annulus were laterally peeled away and the surrounding cartilages were removed exposing the cochlear capsule. The cochlear capsules were bit off in small pieces from the oval window to the apex or shucked off from the base integrally. The stria vascularis and spiral ligament were stripped away as a single piece from the base to the apex, and the OC was separated away from the modiolus.

### OC explant culture

The OC explants were incubated in 4-well culture dishes (4 × 1.9 cm^2^, Nunc, Wiesbaden, Germany) containing 500 μl medium. For different further use, OC explants were cultured in two different ways. For subsequent reverse transcription polymerase chain reaction (RT-PCR) and enzyme-linked immunosorbent assay (ELISA), whole OC tissue was incubated free-floating in the medium. For subsequent histological use, the OC was cut into three parts consisting of apical, middle and basal turns, and then the OC segments were positioned on the bottom of dishes as a flat surface preparation and were held in place by the surface tension of the culture medium. The culture medium consisted of DMEM/F12 (1:1) (Gibco, Karlsruhe, Germany) supplemented with 10% FBS (Biochrom AG, Berlin, Germany), 0.6% glucose, 2 μl/ml insulin-transferrin-Na-Selenit-Mix (Roche Diagnostics GmbH, Mannheim, Germany), 24 ng/ml recombinant human insulin-like growth factor-I (rhIGF-I, R&D Systems, Wiesbaden-Nordenstadt, Germany), and 100 U/ml penicillin (Grünenthal GmbH, Aachen, Germany). The explants were placed in an incubator (SANYO MCO-16AIC, 37°C, 5% CO_2_) for 24 h to condition them before further treatments.

### GA and gentamicin treatment

GA (Cat. # Ant-g1, Invivogen, Toulouse, France) was dissolved in DMSO to make 1 mg/ml stock solutions. After initial 24 h of culture, the medium was exchanged with new medium containing a specific concentration of GA (0.5, 1 or 2 μM) for up to 24 h. Then, OC explants were fixed for staining or lysed for RNA isolation and protein extraction.

Gentamicin sulfate (G1264, CAS # 1405-41-0, Sigma-Aldrich, Munich, Germany) was dissolved in distilled water to prepare 50 mM stock solution and diluted into culture medium at final concentration of 500 μM. After initial 24 h, OC explants were incubated in culture medium containing 2 μM GA for 4 h and then exposed to 500 μM gentamicin for 24 h, or were treated simultaneously with GA and gentamicin for 24 h.

Total RNA samples were isolated from OC cultures using the RNeasy Mini Kit (Qiagen GmbH, Hilden, Germany) and on-column DNase digested with RNase-free DNase Set (Qiagen GmbH, Hilden, Germany) according to the manufacturer's instructions. The isolated RNA was quantified spectrophotometrically with Ribogreen^® ^RNA Quantitation Reagent (Molecular Probes, Göttingen, Germany) and then stored at -80°C until needed.

### cDNA preparation

First-strand cDNA was synthesized from 100 ng of total RNA in a thermocycler (Perkin Elmer-applied biosystems thermal cycler 9600, Foster City, USA). The RNA was denatured by heating at 70°C for 5 min and quickly cooled at 4°C. The reaction mixture contained 0.5 mM dNTP Mix (Invitrogen GmbH, Karlsruhe, Germany), 3.8 μM Oligo(dT) (Biotez, Berlin, Germany), 26 U RNasin (Promega Co., Madison, WI, USA) and 25 U MMLV Reverse Transcriptase (Promega Co., Madison, WI, USA) in a final volume of 20 μl of MMLV Reaction Buffer (Promega Co., Madison, WI, USA). The RT reaction was performed at 42°C for 60 min followed by enzyme inactivation at 95°C for 5 min and cooling at 4°C. To exclude cross-reaction of PCR primers with containing DNA in the following PCR experiment, negative RT controls containing all reverse transcription components, including RNA samples, were prepared by carrying out the reaction in the absence of MMLV RT. In addition, another negative control containing nuclease-free water instead of RNA was set up.

### Real-time quantitative PCR

The Master Mix (containing FastStart Taq DNA Polymerase, reaction buffer, SYBR Green I dye and MgCl_2_) for real-time quantitative PCR (qPCR) was prepared from solution 1a (Enzyme) and solution 1b (Reaction Mix) (LightCycler FastStart DNA Masterplus SYBR Green I, Roche Dianostics GmbH, Penzberg, Germany) according to the manufacturer's instructions. The PCR mixture contained 2 μl of cDNA, 10 pmol each reverse and forward specific primers (BioTez, Berlin, Germany), 4 μl Master Mix and PCR degree water in a final volume of 20 μl. A control PCR reaction, which involved PCR master mix and the primers, but no cDNA template, was used as a blank PCR reaction. Housekeeping gene, encoding ribosomal protein S16 (rS16) was used as internal control. The sequence of primers used in this study was as follows: rS16 (forward GGG TCC GCT GCA GTC CGT TC and reverse CGT GCG CGG CTC GAT CAT CT); HSP70 (*Hsp70-2*) (forward 5'-ACC AGG ACA CTG TTG AGT TC-3' and reverse 5'-ACT CAT CTC CGA GTT CAC AC-3'). PCR was initiated by preincubation at 95°C for 10 min followed by 35 cycles consisting of denaturation at 95°C for 10 sec, annealing at 65-70°C for 10 sec and extension at 72°C for 10-15 sec. Melting curves were obtained by final incubation from 60 to 95°C with a heating rate of 0.1°C/s and ended with cooling down to 4°C in LightCycler System 2.0 (Roche Diagnostics, Basel, Switzerland).

To visualize amplicons, 2 μl of each PCR product was separated by electrophoresis on a 2-3% agarose gel (Gibco BRL, Life Technologies, Paisley, Scotland) stained by GelStar^® ^Nuleic Acid Gel Stain (Cambrex Bio Science, Rockland, USA). The amplicons were visualized under ultraviolet light using a Syngene-Gene Genius imaging system (Synoptics Inc., USA), and the product size were confirmed by comparison with DNA ladder Marker V (Roche Diagnostics, Mannheim, Germany).

Threshold cycle (*Ct*) values acquired in real-time qPCR were normalized to rS16 that served as an endogenous reference and calibrated to the control. Relative expression level (fold change) of the target genes in each experimental sample was calculated using 2^-ΔΔ*C*t ^method [21], where Δ*C*t = *C*t (target gene) - *C*t (reference gene) and ΔΔ*C*t = Δ*C*t (treated) - Δ*C*t (control).

### ELISA

The ELISA kit, Human/Mouse/Rat Total HSP70 DuoSet^® ^IC (Cat. # DYC1663-5, R&D Systems, Wiesbaden-Nordenstadt, Germany), was used to quantify HSP70 protein expression in OC explants. Free-floating OC explants were lysed and the concentration of HSP70 protein in lysates was measured according to the manufacturer's instructions. HSP70 concentration in OC lysates obtained from ELISA was normalized to total protein concentration. Relative expression level of HSP70 in each experimental sample was calculated as picogram of HSP70 per 1 μg of total protein. Six OCs were used per each time point.

### Immunofluorescence

#### HSP70 staining

OC explants were fixed in 4% paraformaldehyde in 0.1 M phosphate-buffered solution (PBS) at room temperature for 30 min. Next, the fragments were washed two times with PBS and permeabilized with 0.2% Triton X-100 in PBS for 30 min. After two washes in PBS, the fragments were incubated in blocking solution (0.8% goat serum, 0.4% Triton and 2% bovine serum albumin in PBS) at room temperature for 3 h and then incubated overnight at 4°C with mouse anti-HSP70 monoclonal antibody (Cat. # SPA-810, Stressgen Bioreagents, Ann Arbor, USA) (1:200 dilution in blocking solution, 5 μg/ml). In the negative control samples, mouse anti-HSP70 monoclonal antibody was substituted with an isotype control (mouse IgG_1_, Dianova, Hamburg, Germany) (1:40 dilution in blocking, 5 μg/ml). The fragments were washed three times in PBS, incubated for 3 h at room temperature with goat anti-mouse IgG conjugated with fluorescence isothiocyanate (FITC) (Dianova, Hamburg, Germany) (1:200 dilution in blocking solution, 7.5 μg/ml) and washed three times in PBS. The fragments were mounted on glass slides in Prolong Gold^® ^antifade reagent (P36930, Molecular Probes (Invitrogen, Karlsruhe, Germany) and examined using a confocal microscope (Leica TCS SPE, Wetzlar, Germany).

#### Hair cell quantification

OC explants were fixed for 30 min in 4% paraformaldehyde in 0.1 M PBS at room temperature. Then, the fragments were washed two times with PBS and permeabilized with 0.2% Triton X-100 in PBS for 30 min. The fragments were washed two times with PBS and incubated with 5 μg/ml solution of phalloidin conjugated with tetramethyl rhodamine isothiocyanate (TRITC) (P1951, Sigma-Aldrich, Munich, Germany) at room temperature for 30 min. After washes with PBS, the fragments were mounted with mounting medium containing 1,4-diazabicyclo[2.2.2]octane (DABCO) (D2522, Sigma-Aldrich, Munich, Germany).

The OC fragments were examined under a fluorescence microscope (Leica DMIL, Wetzlar, Germany) with filters appropriate for TRITC (excitation: 544 nm, emission: 572 nm). There are three rows of OHCs and one row of IHCs along the whole OC. The hair cell numbers were counted over a longitudinal distance of 100 μm in five separated regions of each cochlear part (magnification 400×). Cells were considered missing when there was a gap in the normal arrays and no stereocilia or cuticular plates were to be seen. A mean value was calculated for each explant and at least four explants were used for each experimental condition. The fragments were photographed with a digital camera (Canon PowerShot S40). The contrast and brightness of images were adjusted by using Adobe Photoshop (version 9.0) software.

### Statistical analyses

Means ± standard errors of the mean (SEM) were calculated for all parameters measured. The effect of GA on hair cell viability, HSP70 induction or gentamicin-induced hair cell loss were tested by two-way analysis of variance (ANOVA) followed by Scheffé's post-hoc test. To analyze the effect of GA on gentamicin-induced hair cell loss, the codes of the grouping variable (controls, gentamicin, GA before gentamicin, and GA and gentamicin simultaneously) were selected altogether and in pairs. A p-value of less than 0.05 was considered statistically significant. All statistical test and graphics were made using the software package Statistica 7.1 (StatSoft).

## Results

### The effect of geldanamycin on hair cells

**Figure 1:**
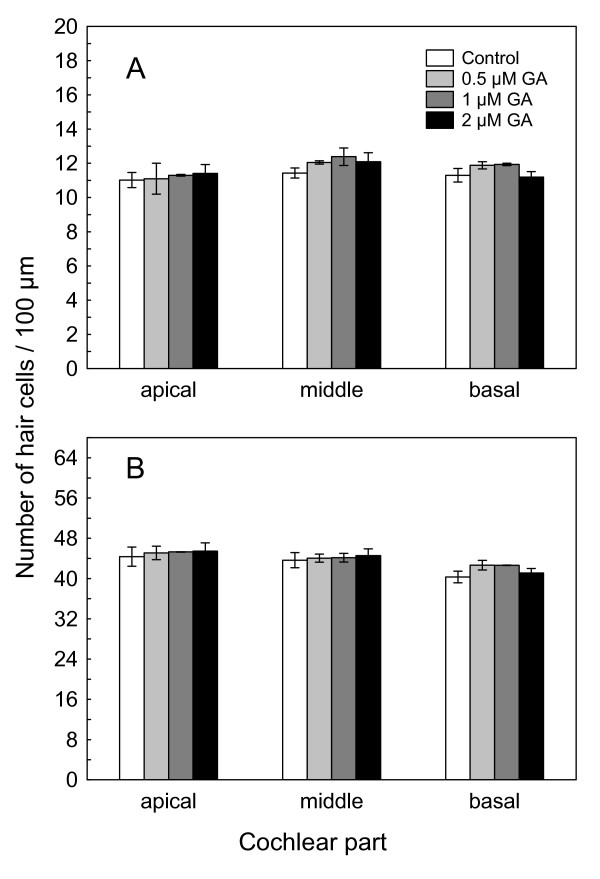
**Geldanamycin does not cause hair cell loss**. Number of hair cells per 100 μm length of the apical, middle and basal parts of the OC explants from controls and GA-treated samples. (A) IHCs; (B) OHCs. Shown are means ± SEM (control and 2 μM GA: n = 6; 0.5 and 1 μM GA: n = 4).

**Figure 2:**
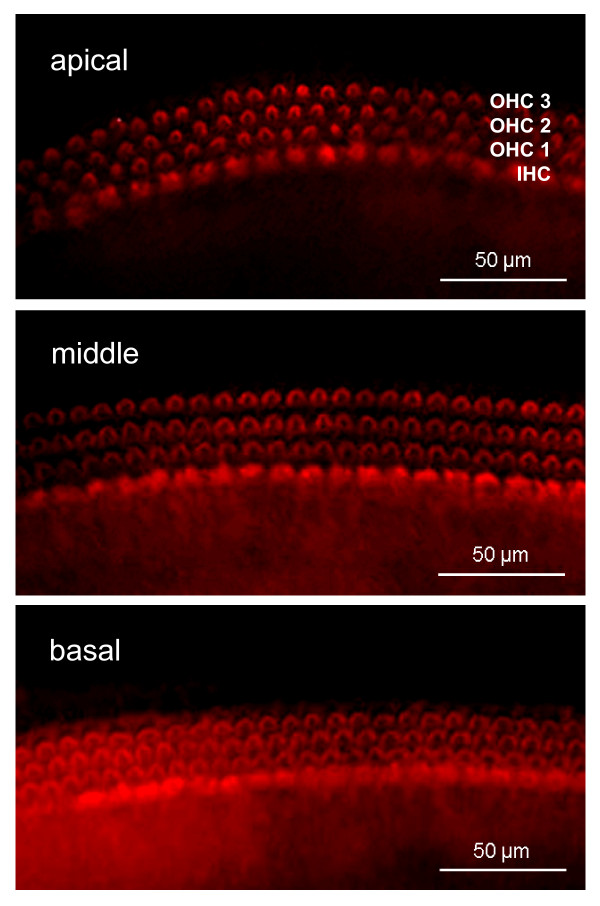
**Geldanamycin does not cause morphological changes in hair cells**. Shown are representative epifluorescence micrographs of phalloidin-TRITC-labeled hair cells in OC cultures treated with 2 μM GA for 24 h.

To determine if GA is safe for use in OC explant cultures and has no cytotoxic effects on hair cells, we treated OC explants for 24 h with GA at varying concentration, which we chose based on the literature [16,18]. Next, using phalloidin staining and hair cell counting, we determined the viability of hair cells. Figure 1 shows the scores of inner and outer hair cells in the OC explants treated with 0.5, 1 and 2 μM GA *versus *untreated explants (control). We have found that GA does not affect the numbers of IHCs (Fig. 1A) and OHCs (Fig. 1B) in apical, middle or basal part of OC, as compared to the untreated controls (two-way ANOVA). The epifluorescence micrographs in Figure 2 show OC explants treated with 2 μM GA for 24 h and then stained with phalloidin-TRITC to label the actin filaments. Three orderly rows of OHCs and one row of IHCs were seen, and the morphology of hair cells was not affected.

Taken together, these results suggest that GA at the concentration between 0.5 μM and 2 μM is not toxic to hair cells in the OC explants. For all further experiments, we have chosen the GA concentration of 2 μM.

### Geldanamycin induces HSP70 production in the OC explants

To determine whether GA induces HSP70 expression in OC and to find out its cellular localization, we treated the explant cultures with 2 μM GA for 24 h and quantified HSP70 expression at mRNA and protein levels by RT-PCR and ELISA, respectively.

**Figure 3:**
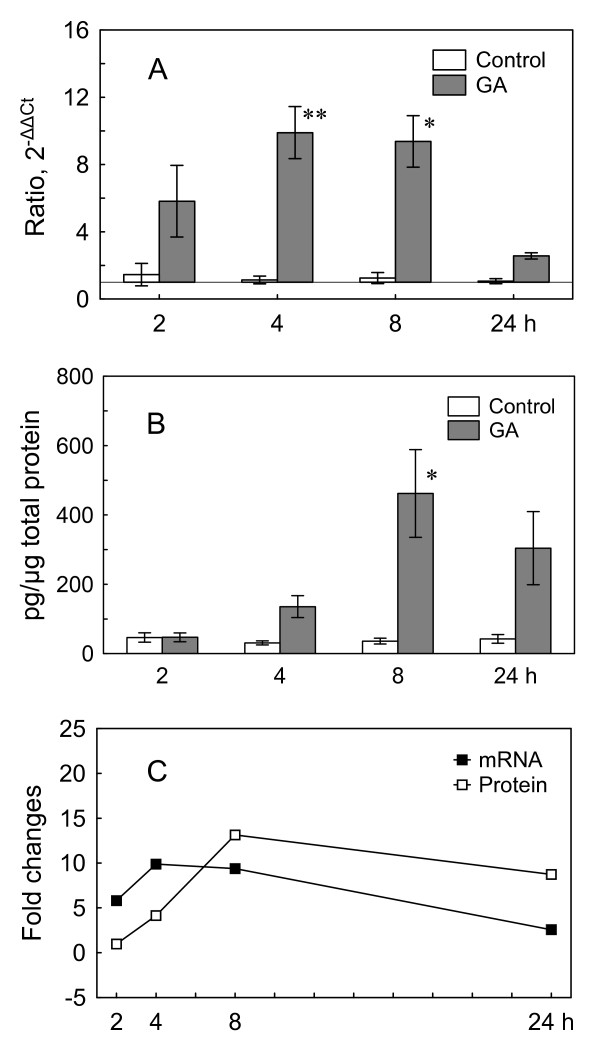
**Geldanamycin induces HSP70 mRNA and protein production in the OC explants**. Time course of HSP70 mRNA and protein expression in OC explants during 2 μM GA treatment. (A) Time course of HSP70 mRNA relative expression (n = 7 each). (B) Time course of HSP70 protein expression (relative to total protein) (n = 6 each). (C) Comparison of time course of HSP70 mRNA and protein expression (fold changes). Shown are means ± SEM. */**p < 0.01/0.001 *vs*. controls.

First, we investigated the time course of HSP70 mRNA expression in OC explants induced by GA. Two-way ANOVA indicated significant differences in the HSP70 mRNA expression between GA-treated groups and controls (p < 0.00001) as well as during different times after induction (p < 0.01). Post-hoc analysis (Scheffé test) revealed, that induction of HSP70 mRNA (Fig. 3A) became significant after 4 h of GA treatment (average 9.9-fold increase against untreated control, SEM = 1.5) and 8 h of treatment (average 9.4-fold increase against control, SEM = 1.5).

Second, we performed ELISA with the GA-treated and untreated OC explant lysates. Two-way ANOVA demonstrated significant differences in the HSP70 concentration between GA-treated groups and controls (p < 0.001) as well as between different times (p < 0.01). Post-hoc analysis (Scheffé test) revealed, that after 8 h of treatment with GA, the concentration of HSP70 rose significantly (Fig. 3B) and reached 462 pg of HSP70/μg of total protein (SEM = 126 pg/μg) as compared to the untreated control (36 pg of HSP70/μg of total protein, SEM = 8 pg/μg).

The concentration of HSP70 relative to total protein concentration in GA-treated explants was normalized to the corresponding controls and presented as a fold change together with the mRNA ratio (Fig 3C). The induction of HSP70 on protein level followed the induction on mRNA level with a 2 h delay (p < 0.05, interaction effect, two-way ANOVA).

**Figure 4:**
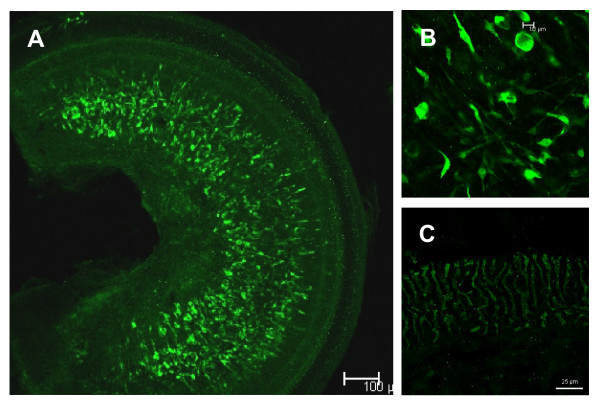
**In the untreated OC explants, HSP70 localizes to limbal fibrocytes and interdental cells**. Shown are the representative laser confocal micrographs of untreated OC cultures stained with a monoclonal antibody against HSP70 followed by FITC-conjugated secondary antibody. (A) the entire explant (basal part); (B) HSP70-positive fibrocytes in spiral limbus; (C) interdental limbal cells.

**Figure 5:**
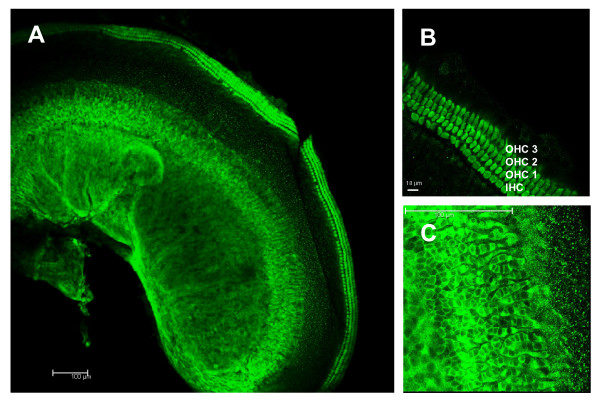
**Geldanamycin-induced HSP70 localizes to hair cells and interdental cells**. Shown are the representative laser confocal micrographs of GA-treated OC cultures stained with a monoclonal antibody against HSP70 followed by FITC-conjugated secondary antibody. (A) the entire explant (basal part); (B) HSP70-positive IHCs and OHCs; (C) HSP70-positive interdental cells.

To localize the HSP70 protein induced by GA in the OC explants, we treated OC explants with 2 μM GA for 8 h (the time point where HSP70 protein expression was highest), fixed and stained the specimens with anti-HSP70 monoclonal antibody. In the untreated OC explants, we observed some HSP70 immunoreactivity in spiral limbus (Fig. 4A), in particular in the fibroblasts (Fig. 4B) and to lesser extend in the interdental cells (Fig. 4C). After 8 h of treatment with GA, OC explants were strongly HSP70-positive (Fig. 5A), in particular OHCs and IHCs (Fig. 5B) and limbal interdental cells (Fig. 5C). There was no HSP70 immunoreactivity in the OC explants where addition of primary anti-HSP70-antibody was substituted with an isotype control.

### Effect of Geldanamycin on gentamicin-induced hair cell loss

**Figure 6:**
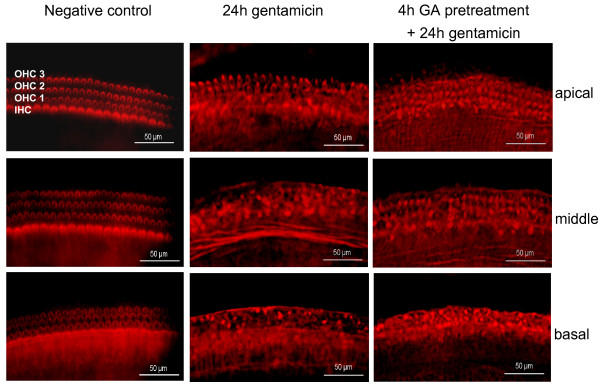
**Geldanamycin protects hair cells against gentamicin-induced toxicity**. Representative epifluorescence micrographs of phalloidin-TRITC-labeled hair cells in untreated OC explants (negative control), treated with gentamicin (500 μM) for 24 h or pretreated with GA for 4 h prior to gentamicin exposure.

Exposure to gentamicin for 24 h led to a severe loss of OHCs in the apical, middle and basal parts of OC explants, as compared to untreated controls (Fig. 6). The stereocilia bundles were missing on most of remaining OHCs.

To study the influence of GA on gentamicin-induced toxicity, we subjected the OC explants to one of the following treatment protocols:

A) 4 h pretreatment with GA (2 μM) prior to 24 h gentamicin treatment (500 μM),

B) simultaneous treatment with GA (2 μM) and gentamicin (500 μM) for 24 h, followed by staining with phalloidin-TRITC to visualize hair cells and scoring under fluorescence microscope.

**Figure 7:**
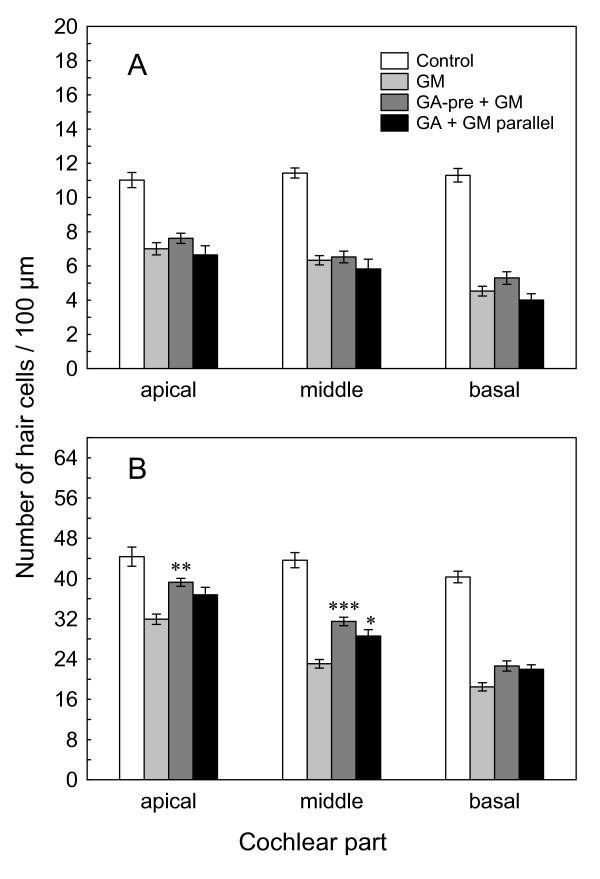
**A) Geldanamycin has no effect on IHC survival during the gentamicin-induced ototoxicity**. Number of IHCs per 100 μm length of the apical, middle and basal parts of the OC explants counted in (1) controls (n = 6), (2) gentamicin-treated (500 μM) samples (GM, n = 15), (3) 4 h of GA (2 μM) pretreatment prior to 24 h of gentamicin (500 μM) treatment (GA-pre + GM, n = 8), and (4) concurrent treatment with GA (2 μM) and gentamicin (500 μM) for 24 h (GA + GM parallel, n = 9). Shown are the means ± SEM. **B) Geldanamycin protects OHC in apical and middle OC parts from the gentamicin-induced ototoxicity**. Number of OHCs per 100 μm length of the apical, middle and basal parts of the OC explants counted in: (1) controls (n = 6), (2) gentamicin-treated (500 μM) samples (GM, n = 15), (3) 4 h of GA (2 μM) pretreatment prior to 24 h of gentamicin (500 μM) treatment (GA-pre + GM, n = 8), and (4) concurrent treatment with GA (2 μM) and gentamicin (500 μM) for 24 h (GA + GM parallel, n = 9). Shown are the means ± SEM. */**/***p < 0.05/0.01/0.001 *vs*. gentamicin-treated samples.

In the OC explants pretreated with GA prior to addition of gentamicin, the loss of OHC was reduced in apical and middle parts of OC, compared to the OHC loss in explants treated with gentamicin alone (Fig. 6). Hair cell scores confirmed this observation. Gentamicin induced a loss of 28%, 47% and 54% of OHCs in the apical, middle and basal part, respectively. Pretreatment of the OC explants with GA significantly reduced this damage (p < 0.00001, two-way ANOVA). Post-hoc analysis (Scheffé test) confirmed that the number of surviving OHCs was higher in the GA-pretreated apical part (39.3 ± 0.8) than in gentamicin-treated only (31.9 ± 1.0), p < 0.001. Similarly, in the middle part, pretreatment with GA increased the number of surviving OHC (31.5 ± 0.8) as compared to gentamicin alone (23.1 ± 0.8), p < 0.0001 (Fig. 7B). However, there was no statistically significant effect of GA pretreatment in the highly damaged basal part of OC.

In the OC explants that were simultaneously treated with GA and gentamicin, the OHC loss in the whole OC was also significantly lower than with gentamicin alone (p < 0.0001, two-way ANOVA). However, the post-hoc test indicated protective effect of GA on OHCs only in the middle cochlear part (p < 0.05). The number of surviving OHCs was 28.6 ± 1.3 in the GA-gentamicin-treated group and 23.1 ± 0.8 in gentamicin treatment alone. These results suggest that the pretreatment of OC explants with GA significantly inhibited loss of OHCs caused by gentamicin.

Gentamicin produced not only a loss of OHCs but also of IHCs (p < 0.001 *vs*. controls). Surprisingly, we did not observe significant protective effect of GA on gentamicin-induced IHC loss (Fig. 7A).

## Discussion

The inner ear is subjected to various types of stress such as aging, acoustic trauma and ototoxic drugs, which can result in cell damage and/or death. Sensory hair cells are extremely susceptible to injury. In contrast to lower species (fish, bird, etc.), mammals cannot regenerate hair cells [22]. Hair cell loss results in an acquired permanent hearing loss that, to date is incurable [23]. Therefore, hair cells are an important target for protective interventions.

One of the substances protecting some cells from death is GA [17]. The implicated mechanism of protection is *via *induction of HSP70 synthesis. In present work, we found that GA induces production of HSP70 on the mRNA and protein level and that HSP70 localizes to the hair cells. We have also unequivocally demonstrated that the pretreatment with GA protects the OHCs but not IHCs in apical and middle parts of OC explants from gentamicin toxicity.

Because of the very high sensitivity of the cochlea to ototoxic substances (especially the basal turn), we first tested GA for its possible toxic properties in this study. Although GA was shown to be toxic to some cells, even at a relative low concentration (0.1 μg/ml or 0.18 μM) [24], in the present study treatment of OC explants for 24 h with 2 μM GA did not cause hair cell damage. Next, we demonstrated that GA up-regulates the expression of HSP70 in OC explants, on the transcriptional and translational levels. The up-regulation of HSP70 mRNA preceded that of HSP70 protein, which suggests that the up-regulation of HSP70 took place on transcriptional level and the increase of HSP70 protein was a secondary process. HSP70 protein expression in OC began within 4 h of treatment with GA and persisted at relative high level until 24 h, corroborating temporal expression pattern seen in previous studies with hippocampal cell line [25] and visual sensory cells [18].

Fluorescence microscopy indicated that GA induced HSP70 mainly in OHCs, IHCs and in interdental cells of spiral limbus, but not in spiral ganglion cell bodies or nerve fibers. This localization pattern of HSP70 protein is similar to that of HSF1 reported by Fairfield et al. [5], except for the negative expression in spiral ganglion. It is also consistent with that of HSP70 mRNA induced by heat shock [26]. However, our results are in contrast to another report showing that HSP70 protein induced by heat shock was present in all cochlear structures except OC [6]. This inconsistency may be due to different stimuli (heat *vs*. GA) or, even more likely, due to different experimental models used (animal *vs*. OC explant).

To damage the hair cells, we used gentamicin and observed more severe hair cell damage in basal part than in apical or middle parts, consistent with previous *in vitro *studies in mice and rat OC explants [27,28]. The question "*why *is gentamicin ototoxic?" was asked by many researches, but it remains open. The major proposed ototoxic mechanism is generation of free radicals. Overproduction of reactive oxygen species and the resulting red-ox imbalance have been demonstrated to be the important mechanism of gentamicin ototoxicity. Priuska and Schacht have found that gentamicin was able to accelerate the formation of free radicals in the presence of iron salts [29]. There is increasing evidence that hair cells damaged by gentamicin die by an active cell death - apoptosis. Gentamicin treatment has been shown to result in chromatin condensation and DNA fragmentation, hallmarkers of apoptosis [30,31]. Moreover, cytochrome *c *release from the mitochondria into the cytoplasm and activation of caspase-8, caspase-9 and caspase-3 were observed in gentamicin-damaged hair cells, suggesting that auditory hair cell death is a result of gentamicin exposure in a caspase-dependent pathway [31,32]. In addition to caspase-mediated apoptosis, the c-Jun N-terminal kinase (JNK) apoptotic pathway was also implicated in gentamicin ototoxicity. It was demonstrated that gentamicin activated JNK-dependent apoptotic pathway in IHCs *in vivo *[33]. Interestingly, the most recent research from the laboratory of Sha demonstrated that in the OC explants isolated from mice, gentamicin induces histone deacetylation and in this way induces cell death [34]. Gentamicin was reported to induce *de novo *production of HSP70 in the renal cell line [35]. However, the synthesis pick of HSP70 was observed relatively late after administration of gentamicin (72 h), in which time the damage to the cochlear hair cells is very extensive, and was related to by authors as a marker of renal injury.

We found that GA partially attenuates gentamicin-induced hair cell loss. The most tempting interpretation on this finding is that the protective effect of GA against gentamicin-induced hair cell loss is the consequent cytoprotective action of HSP70 induced by GA. Harrison et al. proposed a pathway of GA-mediated neuroprotection based on their findings, that GA exerts protection by destabilization of the Hsp90-HSF1 complex, nuclear translocation of HSF1, activation of the Hsp70 promoter and transcriptional up-regulation of HSP70 [36]. Protective effects of HSP70 are attributed to its critical function as a molecular chaperone. This function involves refolding of misfolded and thus toxic proteins in cytoplasm and reducing protein aggregation. Furthermore, previous studies have demonstrated that HSP70 inhibits apoptosis in a variety of systems by interfering with various apoptotic-signaling cascades that were indicated to be involved in mechanism of ototoxicity. HSP70 was shown to strongly inhibit JNK activation [37] and to reduce mitochondrial cytochrome *c *release to cytosol [38]. In addition, HSP70 prevented the recruitment of pro-caspase-9 to the apoptosome complex, thus blocking the assembly of a functional apoptosome and consequent activation of caspase-9 [1]. Lastly, HSP70 was suggested to inhibit processing of caspase-3 [37] and to inhibit cell death even after caspase-3 has been activated [39].

We observed the protective effect of GA in apical and middle parts of OC explants exposed to gentamicin pretreated with GA for 4 h. At that time, HSP70 mRNA had reached significantly high level in the OC explants, followed by protein production. However, when OC explants were concurrently treated with gentamicin and GA, the protective effect of GA was small and targeted only to the middle part of OC. These results are at present encouraging for the protection but not for the treatment of gentamicin-induced ototoxicity. It has already been demonstrated that GA provided post-exposure neuroprotection in HT22 hippocampal cell line against glutamate-induced oxidative toxicity [25]. This encourages further investigations in order to define the therapeutic window for GA.

Even though HSP70 expression was induced by GA in both OHCs and IHCs, and despite the fact that OHCs and IHCs appeared to be equally susceptible to gentamicin, *only *OHCs were protected by GA. These results suggest that the mechanism of OHC and IHC loss may differ between these two cell types and that in the OHCs but not in IHCs, the ototoxic effect of gentamicin may be inhibited by GA-induced HSP70. It is also possible that other than HSP70 targets of GA mediate the protection. For instance, GA not only increased the expression of HSP70 but also the expression of HSP90 and HSP27 in COS-1 cells [40] and renal cells [36]. HSP90 and HSP27 have been shown to suppress the aggregation of various proteins and facilitate degrading unwanted and/or harmful proteins, hence act as general protective chaperones [41,42]. They also can inhibit apoptosis by preventing the assembly of apoptosome [42,43]. It has been demonstrated that HSP90 and HSP27 were induced in IHCs by heat shock [10,13]. It is unknown whether HSP90 and HSP27 were induced by GA in the present study, which will be determined in further investigations.

Nevertheless, based on the present results, the protective effect of GA against ototoxic hair cell death may be attributed to the actions of HSP70 induced by GA. Further investigations are needed to reveal the exact molecular mechanisms underlying the otoprotective role of GA, which may provide insights for therapeutic approaches aimed at preventing hair cell damage induced by various ototoxic agents, or even other deleterious stresses.

The administration of GA in clinical settings is limited due to its high hepatotoxicity [24]. In addition, GA has shown to be incapable of crossing the blood brain barrier [44], which is very similar to blood labyrinth barrier [45]. These disadvantages led to the development of GA analogues. 17-Allylamino-17-demethoxygeldanamycin (17-AAG) is an analogue chemically derived from GA. 17-AAG is considered less hepatotoxic and more stable than GA and has been shown to cross the blood brain barrier [46]. It is currently in early stage clinical trials as a chemotherapeutic agent [47]. Another analogue, 17-Dimethylaminoethylamino-17-demethoxygeldanamycin (17-DMAG) is more water-soluble than 17-AAG and has excellent bioavailability, thus is more practicable in preclinical models [48]. 17-AAG and 17-DMAG have been indicated to induce HSP70 in a variety of cells and provided some corresponding protective effects [36,48-50]. Although there is no evidence so far supporting otoprotective effect of these two GA analogues, 17-AAG and 17-DMAG may be supposed to have protective roles in inner ear, which would be validated in future studies.

## Conclusion

In conclusion, we show for the first time that GA induces transcription and translation of HSP70 in the OC explants. In addition, we demonstrate the protective effect of GA against gentamicin ototoxicity in the OHCs but not the IHCs. GA may provide insights for therapeutic approaches aimed at preventing and ameliorating the extent of acquired hearing loss.

## Abbreviations

17-AAG: 17-Allylamino-17-demethoxygeldanamycin; ANOVA: analysis of variance; BSG: buffered saline glucose solution; *C*t: threshold cycle; 17-DMAG: 17-Dimethylaminoethylamino-17-demethoxygeldanamycin; ELISA: enzyme-linked immunosorbent assay; GA: geldanamycin; HSF: heat shock factor; HSP: heat shock protein; IHCs: inner hair cells; JNK: c-Jun N-terminal kinase; OC: organ of Corti; OHCs: outer hair cells; PBS: phosphate-buffered solution; qPCR: real-time quantitative polymerase chain reaction; rS16: ribosomal protein S16; RT-PCR: reverse transcription polymerase chain reaction; SEM: standard error of the means; TRITC: tetramethyl rhodamine isothiocyanate.

## Competing interests

The authors declare that they have no competing interests.

## Authors' contributions

YY collected, analyzed and interpreted the data and drafted the manuscript. AJS was responsible for conception and design of the project, interpreted the data and critically revised the manuscript. HH analyzed and interpreted the data and critically revised the manuscript. BM was responsible for conception and design of the project, interpretation of the data and drafting the manuscript.
